# Intraocular Coinfection by *Toxoplasma gondii* and EBV Possibly Transmitted Through Unpasteurized Goat Milk in an Immunocompetent Patient: A Case Report

**DOI:** 10.3390/pathogens14121222

**Published:** 2025-11-30

**Authors:** Juanita Cardona-López, Francisco J. Rodríguez, Ricardo Igua, Alejandra de-la-Torre

**Affiliations:** 1 Neuroscience (NEUROS) Research Group, Neurovitae Research Center, Institute of Translational Medicine (IMT), Escuela de Medicina y Ciencias de la Salud, Universidad del Rosario, Bogotá 111221, Colombia; juanitacardona.jc@gmail.com; 2 Fundación Oftalmológica Nacional (FUNDONAL), Escuela de Medicina y Ciencias de la Salud, Universidad del Rosario, Bogotá 111221, Colombia; fjrodriguez@fon.org.co (F.J.R.); ricardo.igua@urosario.edu.co (R.I.)

**Keywords:** atypical ocular toxoplasmosis, Epstein–Barr virus, coinfection, unpasteurized goat milk, refractory panuveitis, vitreous hemorrhage

## Abstract

*Toxoplasma gondii* is the most common infectious cause of posterior uveitis in immunocompetent adults. While the parasite is typically acquired through ingestion of undercooked meat or contaminated food and water, unpasteurized goat milk has been identified as a less frequent but plausible source of infection. Coinfections in ocular toxoplasmosis are rare, and the role of Epstein–Barr virus (EBV) in these coinfections remains poorly understood. We report the case of a 70-year-old immunocompetent male presenting with severe, refractory panuveitis in the left eye. Initial serologic testing confirmed acquired *Toxoplasma gondii* infection, and treatment was initiated with systemic antimicrobials and corticosteroids. Intraocular inflammation persisted despite sequential therapy with trimethoprim–sulfamethoxazole, clindamycin, and azithromycin, eventually requiring pars plana vitrectomy with intravitreal clindamycin and dexamethasone due to non-clearing vitreous hemorrhage. Vitreous PCR testing revealed intraocular concurrent detection of EBV DNA, prompting combined antimicrobial and antiviral therapy. Epidemiological history revealed recent consumption of unpasteurized goat milk, suggesting a potential oral transmission route for *Toxoplasma gondii*. Although visual acuity improved following surgical intervention and targeted therapy, it remained markedly compromised due to the severity of the disease. This case illustrates the diagnostic value of multiplex PCR in refractory uveitis, enabling the detection of *Toxoplasma gondii* and the concurrent detection of EBV DNA in an immunocompetent patient. It highlights the importance of early molecular testing and detailed epidemiological assessment, including atypical transmission routes such as unpasteurized goat milk.

## 1. Introduction

*Toxoplasma gondii* is an intracellular protozoan with global distribution, recognized as the leading infectious cause of posterior uveitis in immunocompetent adults [1,2]. It can be transmitted through two main routes: ingestion of tissue cysts, primarily from undercooked or raw meat, and ingestion of oocysts, which may occur through contaminated water, unwashed fruits or vegetables, or direct contact with cat feces [1,2]. Although less frequently, transmission through unpasteurized goat milk has also been reported. A case–control study in the United States identified this route as a significant risk factor for acute *Toxoplasma gondii* infection (adjusted OR 5.09; 95% CI: 1.45–17.80) [3]. While systematic reviews and meta-analyses suggest that raw milk did not reach statistical significance as a global transmission source [4], outbreak reports from previous decades documented contaminated milk with tachyzoites as a relevant source of infection [5,6,7]. Recent reviews and epidemiological guidelines continue to recognize this route as plausible, especially in particular geographic or cultural contexts. Although uncommon, milk-borne transmission remains relevant in atypical presentations [8].

Coinfections in ocular toxoplasmosis are rare and scarcely described in the literature [9]. A cross-sectional study in immunocompetent patients with infectious uveitis, using PCR and the Goldmann–Witmer coefficient, identified coinfections primarily with *Mycobacterium tuberculosis* or herpes simplex virus. No cases of coinfection with *Toxoplasma gondii* and Epstein–Barr virus (EBV) were documented, despite EBV being included in the diagnostic panel. This underscores the utility of multiplex molecular techniques in distinguishing entities with overlapping features, such as ocular toxoplasmosis [9].

Although rare, intraocular infection with EBV has been associated with significant inflammation and severe retinitis, particularly in immunosuppressed individuals [10]. Coinfection with *Toxoplasma gondii* has been reported in immunocompromised patients; however, in immunocompetent individuals, the pathogenic role of EBV remains uncertain, and its coexistence with *Toxoplasma gondii* is considered exceptionally rare [11].

This report describes a severe, refractory case of panuveitis in an immunocompetent patient with intraocular coinfection by *Toxoplasma gondii* and Epstein–Barr virus (EBV), confirmed by means of multiplex PCR of vitreous humor. It highlights three uncommon and insufficiently documented aspects: probable transmission through unpasteurized goat milk, the concurrent detection of EBV, whose clinical significance in ocular toxoplasmosis remains unclear, and the occurrence of vitreous hemorrhage. Therapeutic refractoriness led to vitrectomy and combined antimicrobial therapy, highlighting the importance of considering viral coinfections and atypical epidemiological exposures in cases with torpid evolution.

## 2. Case Presentation

A 70-year-old immunocompetent male presented with a one-week history of floaters and blurred vision in the left eye (OS). On initial examination, best corrected visual acuity (BCVA) was 20/20 in the right eye (OD) and 20/50 in the OS. Slit-lamp examination showed numerous granulomatous keratic precipitates in Arlt’s triangle and elevated intraocular pressure (IOP) of 38 mmHg in OS (OD: 12 mmHg). Fundus examination of the OS revealed severe vitreous opacities and a temporal active retinochoroidal lesion. Serology for *Toxoplasma gondii* was positive (IgM: 30.5 IU; IgG: 14.5 IU), supporting the diagnosis of panuveitis with secondary ocular hypertension. Treatment with trimethoprim/sulfamethoxazole and clindamycin was initiated.

After three months, persistent inflammation prompted further evaluation. The OS showed mutton fat keratic precipitates and moderate vitritis; media opacity limited OCT imaging, and the fundus remained obscured.

A vitreoretinal specialist reported severe visual loss in OS (light perception). Anterior segment examination disclosed granulomatous keratic precipitates and 2+ cells in the anterior chamber. Dense vitritis precluded detailed visualization of the retina, which appeared attached. Therapy was adjusted to azithromycin, prednisone, trimethoprim–sulfamethoxazole, topical steroids, and cycloplegics.

Seven months after the onset of symptoms, the patient was evaluated by an ocular immunology specialist elsewhere. Aqueous humor PCR was positive for *Toxoplasma gondii* (2200 copies/mL). Although systemic parasitemia quantification was not performed, the evolution of the serologic profile indirectly reflected the decline in parasitic burden. Ten months after presentation, follow-up testing showed *T. gondii*-specific IgM negativity (0.58 IU/mL) and a marked increase in IgG titers (>500 IU/mL), consistent with an active infection that had progressed toward immunological response.

Although anterior segment inflammation had resolved, posterior inflammation persisted with progressive visual decline. Treatment included topical and systemic corticosteroids, azithromycin (1 g on day one, then 500 mg/day), and trimethoprim–sulfamethoxazole. Once inflammation was controlled, combined vitrectomy and cataract surgery were proposed.

Due to poor response to conventional therapy, a diagnosis of atypical panuveitis secondary to refractory ocular toxoplasmosis was made. At our center, a thorough clinical history and review of systems revealed prior consumption of unpasteurized goat milk before symptom onset, followed by gastrointestinal manifestations, raising suspicion of foodborne transmission.

**Figure 1:**
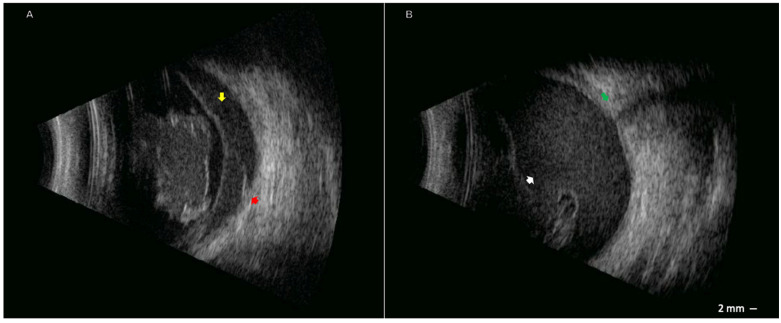
Ocular ultrasound was performed eight months after symptom onset. (**A**) Choroidal thickening (red arrow) and subhyaloid opacities (yellow arrow). (**B**) Dense vitreous opacities (white arrow) with a normal-appearing optic nerve (green arrow).

At our initial examination, BCVA was 20/20 in the OD and light perception in the OS. The OS showed 0.5+ anterior chamber cells, mild flare, and 360° posterior synechiae. There was a severe vitreous hemorrhage, which precluded fundus examination of the OS. An ultrasound of the OS revealed choroidal thickening, subhyaloid and dense vitreous opacities, with a normal-appearing optic nerve and an attached retina (Figure 1).

**Figure 2:**
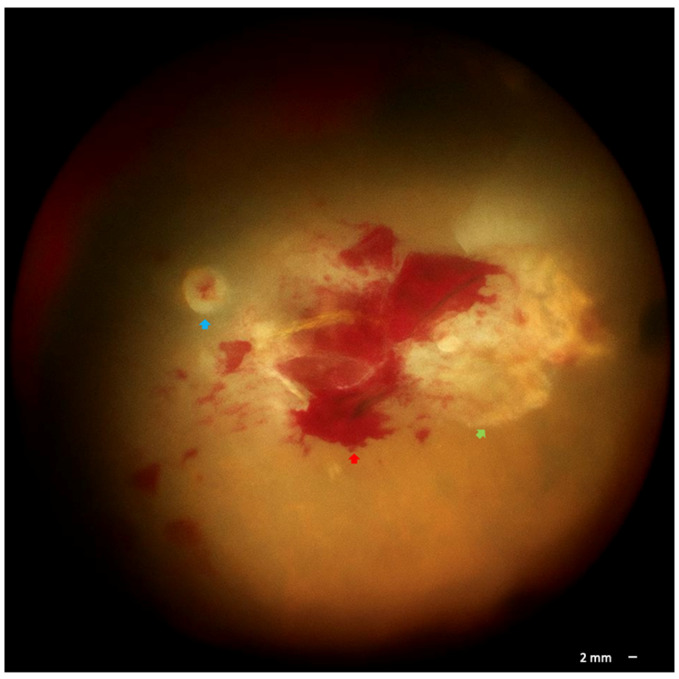
Intraoperative fundus image showing pale optic nerve (blue arrow), retina hemorrhages in the posterior pole (red arrow), severe peripheral ischemia, and non-active temporal scar (green arrow).

Due to therapeutic failure and the presence of a non-clearing vitreous hemorrhage, posterior vitrectomy with intravitreal clindamycin and dexamethasone was performed in the OD. Intraoperative findings included cataract, severe vitreous hemorrhage, retinochoroidal scars involving the macular area, and areas of retinal ischemia (Figure 2). Multiplex PCR of vitreous fluid revealed concurrent detection of EBV DNA. Additionally, repeat serology confirmed markedly elevated *Toxoplasma gondii* IgG (>500 IU/mL) with negative IgM, together with positive EBV IgG (32.7; cutoff > 11) and negative IgM, further supporting the diagnosis of concomitant toxoplasmosis and EBV coinfection. Combined therapy was initiated with pyrimethamine, sulfadiazine, folinic acid, and valacyclovir.

**Figure 3:**
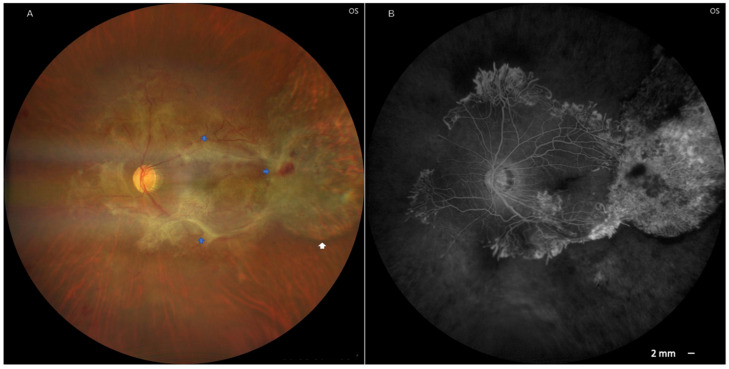
Fluorescein angiography of the left eye was performed one month after surgery. Image (**A**) shows an extramacular retinochoroidal scar (white arrow) and fibrovascular proliferation (blue arrow). In image (**B**), fluorescein angiography shows several ischemic and non-active scars.

At the last follow-up examination, one week after the vitrectomy, the BCVA had improved to 20/400 in the OS. Fundus examination showed inactive scars in the macular area and the temporal aspect of the OS (Figure 3).

## 3. Discussion

Ocular toxoplasmosis is the leading infectious cause of posterior uveitis worldwide. In most cases, it responds adequately to systemic antimicrobial therapy, generally combined with corticosteroids [2,8,12]. However, atypical presentations may occur, including severe panuveitis, extensive retinal vasculitis, diffuse retinal necrosis, and vitreous hemorrhage, even in immunocompetent individuals [2,8,12,13,14]. These cases require an expanded diagnostic approach that includes molecular tests such as PCR on intraocular fluids or intraocular antibody production, since conventional serology has limited specificity in regions with high seroprevalence [2,15].

In this case, intraocular antibody detection or comparative antibody profiling with serum was not performed because the diagnosis was already confirmed by consistent clinical, serologic, and molecular evidence of acute toxoplasmosis. The patient exhibited a characteristic necrotizing retinochoroiditis, together with positive *Toxoplasma gondii*-specific IgM (30.5 IU) and IgG (14.5 IU), and molecular detection of *Toxoplasma gondii* DNA in the aqueous humor sample (2200 copies/mL). This combination of findings provided robust confirmation of active infection, rendering additional intraocular antibody analysis unnecessary. However, determination of local antibody synthesis through the Goldmann–Witmer coefficient or comparative immunoblot remains a valuable diagnostic alternative in cases where molecular assays are unavailable or when differentiation between systemic and intraocular antibody production is required. The Goldmann–Witmer coefficient has also shown utility in diagnosing infectious uveitis with coexistence of *Toxoplasma gondii* and other pathogens [9].

In regions with limited diagnostic resources where molecular tools such as multiplex PCR are not accessible, the diagnosis of ocular toxoplasmosis relies mainly on clinical assessment, supported by conventional serological testing. The characteristic fundus appearance, a necrotizing retinochoroiditis contiguous to a pigmented retinochoroidal scar, often accompanied by mild or moderate vitritis, remains the hallmark of the disease and strongly suggests infection by *Toxoplasma gondii* [16,17]. In routine practice, most specialists establish the diagnosis clinically, usually with serological confirmation, reserving the analysis of intraocular fluid for atypical or diagnostically uncertain cases [16,17].

Serologic evaluation of *Toxoplasma gondii*-specific IgG and IgM antibodies can help differentiate between recent and old infections, while IgG avidity testing may further help determine the infection timeline [18]. Nevertheless, seropositivity alone is insufficient for diagnosis, as background exposure to *Toxoplasma gondii* is high in many populations [18].

When molecular confirmation is not feasible, a favorable clinical response to anti-*Toxoplasma* therapy can further support the diagnosis [16]. Therefore, an integrated evaluation that combines clinical findings, serologic results, and therapeutic response remains essential for accurate diagnosis in settings lacking advanced laboratory infrastructure [16,17,18].

Treatment of these refractory forms is not standardized and may involve multiple regimens, including intravitreal drug administration, particularly indicated in cases with macular (zone 1) involvement, in patients who are refractory to systemic therapy, or when systemic treatment is contraindicated or poorly tolerated. However, current evidence does not support the superiority of any specific regimen [8,12]. The present case is notable for several atypical features: chronic and refractory course, with unilateral panuveitis, the presence of vitreous hemorrhage, therapeutic failure requiring multiple regimens, intraocular detection of EBV, and the need for surgical intervention. Additionally, the patient reported consuming unpasteurized goat milk before symptom onset, which was followed by gastroenteritis, a recognized but uncommon zoonotic transmission route.

While ingesting raw or undercooked meat and drinking untreated water are the most common foodborne sources of *Toxoplasma gondii*, consuming unpasteurized goat milk has been identified as a potential source, particularly in settings where the practice is common or unregulated [19,20,21,22]. Epidemiological studies have reported anti-*Toxoplasma gondii* antibodies in goat milk, supporting the need for surveillance in extensive or semi-intensive goat farming systems [21,22]. Clinical guidelines and meta-analyses recognize this route as infrequent but plausible, especially in endemic regions or vulnerable populations [19,20]. In this case, the patient’s recent history reinforces the plausibility of this oral transmission route in unregulated settings.

Another unusual finding was the detection of EBV in the vitreous fluid by means of multiplex PCR. Although this virus is more frequently reported in immunosuppressed patients, it has also been described in immunocompetent individuals [23]. Viral coinfections in uveitis have been documented, most commonly involving cytomegalovirus or herpesviruses; however, coinfection with *Toxoplasma gondii* and EBV in immunocompetent individuals is exceedingly rare and has only been sporadically reported [24,25].

The use of molecular diagnosis enabled the identification of infectious agents and the adjustment of therapy. EBV has a high global seroprevalence and may be detected intraocularly in healthy individuals or in patients with uveitis, frequently associated with other pathogens [11,23,24]. This virus can modulate ocular immune responses by producing an IL-10 homolog that suppresses effective immunity against pathogens such as *Toxoplasma gondii*, potentially leading to more severe inflammation [9]. Infections by atypical *Toxoplasma gondii* strains, common in our region, promote a Th2-skewed immune profile, reducing cell-mediated immunity and potentially reactivating latent viruses such as EBV [9,10,26].

EBV-infected B lymphocytes have been reported to infiltrate enlarged retinochoroidal lesions, typically in immunosuppressed patients. Although our patient was immunocompetent, the presence of vitreous hemorrhage could have facilitated the entry of B lymphocytes, known EBV reservoirs, into the vitreous humor [11].

Multiplex PCR enables simultaneous detection of multiple pathogens and has demonstrated diagnostic value in atypical or treatment-resistant uveitis [9]. Although EBV detection alone does not imply causality, it has been associated with severe ocular inflammation and therapeutic resistance, potentially justifying antiviral therapy or surgical management in selected cases [8,23]. Based on the current findings, we cannot determine whether the EBV DNA detected in the vitreous sample represents an incidental finding, a causal pathogen, or an amplifier of intraocular inflammation. PCR confirms the presence of viral DNA but does not indicate viral activity; EBV may be present within circulating or resident B lymphocytes or could have been secondarily detected due to vitreous hemorrhage, without necessarily implying active replication or pathogenic involvement. Quantitative PCR helps differentiate latent from active infection based on viral load: in latency, EBV DNA levels are typically <100 copies/10^5^ PBMC and undetectable in plasma [27,28,29], whereas active infection shows >1000 copies/10^5^ PBMC or >500 copies/mL in plasma, reflecting viral replication [30]. Although intraocular thresholds are not validated, these criteria can be extrapolated to ocular fluids and must always be interpreted within the clinical context [29]. Altogether, these findings support the role of broad molecular testing in complex uveitis, even though *Toxoplasma gondii*–EBV concurrent detection of EBV DNA remains an exceptional observation [8,23,24,25]. In this case, another atypical manifestation was vitreous hemorrhage, which may have been caused by retinal neovascularization, a rare complication of ocular toxoplasmosis, potentially triggered by vascular occlusion within toxoplasmic scars, capillary nonperfusion, and adjacent neovascular growth [10,13].

This case highlights the importance of incorporating comprehensive molecular studies into the workup of refractory uveitis, particularly for identifying viral concurrent detection, such as EBV, whose detection in vitreous fluid can influence therapeutic decisions. It also underscores the relevance of detailed epidemiological history, including atypical transmission routes such as unpasteurized goat milk. Integrating clinical, epidemiological, and molecular data allows for accurate diagnosis and tailored management, including surgery when indicated.

## Data Availability

The data supporting the findings of this case report are not publicly available due to patient privacy restrictions. Clinical and laboratory data are fully described within the article, and additional details may be obtained from the corresponding author upon reasonable request.
